# Long-term CD4+ lymphocyte response following HAART initiation in a U.S. Military prospective cohort

**DOI:** 10.1186/1742-6405-8-2

**Published:** 2011-01-18

**Authors:** Alan R Lifson, Elizabeth M Krantz, Lynn E Eberly, Matthew J Dolan, Vincent C Marconi, Amy C Weintrob, Nancy F Crum-Cianflone, Anuradha Ganesan, Patricia L Grambsch, Brian K Agan

**Affiliations:** 1 Division of Epidemiology and Community Health, University of Minnesota, Minneapolis, MN, USA; 2 Division of Biostatistics, University of Minnesota, Minneapolis, MN, USA; 3 Defense Institute for Military Operations, Wilford Hall USAF Medical Center, San Antonio, TX, USA; 4 School of Medicine, Emory University, Atlanta, GA, USA; 5 Infectious Disease, Walter Reed Army Medical Center, Washington, DC, USA; 6 Infectious Disease, Naval Medical Center-San Diego, San Diego, CA, USA; 7 Infectious Disease, National Naval Medical Center, Bethesda, MD, USA; 8 Infectious Disease Clinical Research Program, Uniformed Services University of Health Sciences, Bethesda, MD, USA

## Abstract

**Background:**

Among HIV-infected persons initiating highly active antiretroviral therapy (HAART), early CD4+ lymphocyte count increases are well described. However, whether CD4+ levels continue to increase or plateau after 4-6 years is controversial.

**Methods:**

To address this question and identify other determinants of CD4+ response, we analyzed data for 1,846 persons from a prospective HIV military cohort study who initiated HAART, who had post-HAART CD4+ measurements, and for whom HIV seroconversion (SC) date was estimated.

**Results:**

CD4+ count at HAART initiation was ≤ 200 cells/mm^3 ^for 23%, 201-349 for 31%, 350-499 for 27%, and ≥500 for 19%. The first 6 months post-HAART, the greatest CD4+ increases (93-151 cells) occurred, with lesser increases (22-36 cells/year) through the first four years. Although CD4+ changes for the entire cohort were relatively flat thereafter, HIV viral load (VL) suppressors showed continued increases of 12-16 cells/year. In multivariate analysis adjusting for baseline CD4+ and post-HAART time interval, CD4+ responses were poorer in those with: longer time from HIV SC to HAART start, lower pre-HAART CD4+ nadir, higher pre-HAART VL, and clinical AIDS before HAART (P < 0.05).

**Conclusions:**

Small but positive long-term increases in CD4+ count in virally suppressed patients were observed. CD4+ response to HAART is influenced by multiple factors including duration of preceding HIV infection, and optimized if treatment is started with virally suppressive therapy as early as possible.

## Background

Among those with human immunodeficiency virus (HIV) infection, the CD4+ T-lymphocyte count is the major indicator of immunodeficiency, a main factor in deciding whether to initiate highly active antiretroviral therapy (HAART), and an important parameter in monitoring treatment response [1,2]. Failure to restore a normal CD4+ count following HAART is associated with increased morbidity due to both AIDS and non-AIDS events, as well as increased mortality [3-5].

Studies of the kinetics of CD4+ count response post-HAART indicate that the CD4+ count increases rapidly during the first 3-6 months, in part due to release of memory T-cells from lymphoid tissue, and then increases slowly during the next 3-4 years, reflecting reconstitution of the immune system [6-10]. The magnitude of CD4+ recovery may depend on a variety of factors, including maintenance of virologic suppression, age, and CD4+ count at HAART initiation [1,7,9,11-20].

The question of whether those initiating HAART will continue to increase their CD4+ count after 4-5 years or will plateau has been debated in the literature, and remains unclear. Some studies have suggested that normalization of CD4+ counts in HIV-infected persons can be achieved if viral suppression with HAART can be maintained for a sufficiently long period of time [19]. In one study, after > 5 years on HAART, patients with viral suppression who started at ≤200 cells/mm^3 ^had an adjusted annual increase of 32 cells/mm^3^, attaining an average CD4+ count of 497 cells/mm^3 ^[19]. Another study statistically estimating the CD4+ trajectory concluded that those starting HAART at ≤200 CD4+ cells who remained on therapy would continue to increase through 7 years, although 25% still had ≤350 cells at 7 years [20]. One small study of 16 patients followed for up to 10 years with strict viral control based on HIV RNA detection using ultrasensitive techniques showed continued positive increases in CD4+ counts, although this study represented a small group of highly selected patients [21]

On the other hand, other studies report that the average CD4+ count may level off after 4-6 years following HAART initiation, even among patients with viral suppression [12,13]. Given this leveling off, many patients who start at lower CD4+ counts, even after years on HAART with early CD4+ increases, may fail to reach a normal CD4+ threshold. In one study of those with sustained viral suppression who started HAART at ≤200 CD4+ cells/mm^3^, after 6 years only 42% had ≥ 500 CD4+ cells/mm^3^, and only 12% had >750 cells/mm^3 ^[12]. In another study, 44% of those starting therapy with a CD4+ count <100 cells/mm^3 ^and 25% of those starting HAART with a CD4+ count of 100-200 cells were unable to achieve a CD4+ cell count >500 cells/mm^3 ^over a mean follow-up of seven years, and many did not reach this threshold by year 10 [18].

The important question of the long-term CD4+ count response therefore remains unresolved. This question is especially relevant for those who start HAART at lower CD4+ counts. Despite current recommendations to start HAART at CD4+ counts of 350 cells/mm^3 ^or greater [1,2], the reality is that many patients, even in developed countries, are still being diagnosed and initiate treatment late in the course of their HIV infection [22,23].

An additional methodological challenge in using observational data to evaluate the long-term effect of CD4+ count at HAART initiation on subsequent response is that those starting HAART at lower CD4+ levels may have been infected for longer periods of time. If the post-HAART response is affected by duration of HIV infection, comparing different strata without accounting for the fact that those initiating HAART at lower CD4+ levels may have a longer lead-time can result in biased group comparisons [24].

We were able to address both of these issues by analyzing data from the U.S. Military HIV Natural History Study (NHS) [25]. This prospective cohort of HIV-infected U.S. military personnel has followed some participants for up to twelve years after availability of HAART. Because all active duty personnel are confirmed to be HIV-negative prior to enlistment and undergo routine HIV screening, HIV seroconversion (SC) date can be reliably determined for the majority of members. All cohort members have free access to care and availability of therapy. Data from this cohort were analyzed to determine the long-term CD4+ count trajectory after HAART initiation, as well as the influence of baseline CD4+ count, duration of HIV infection, and other covariates on post-HAART CD4+ response.

## Methods

### Study Cohort and Data Elements

The NHS is an observational prospective cohort study of consenting U.S. military personnel and beneficiaries [25]. Since 1985, routine HIV testing has been used to restrict HIV-infected persons from enlistment. Active duty personnel undergo repeat HIV screening every 1-5 years. Those found HIV-positive after enlistment, plus HIV-positive retirees and dependents of active duty personnel, receive free medical evaluation and ongoing care at military medical centers. Although HIV transmission risk groups are not routinely assessed, injection drug use was not self-reported by any Navy or Marine personnel who seroconverted for HIV during 1997-8 [26]. More recently, hepatitis C prevalence of only 3% was reported for evaluable subjects in this cohort [27], consistent with low injection drug use.

Since 1986, the NHS has enrolled 5,091 HIV-positive participants; NHS protocol is for patients to be seen every six months at one of seven participating military medical centers. Data collected include demographics, medical histories including medication use, and laboratory measures including CD4+ count. In 1996, HIV viral load (VL) became available to the study.

This analysis was limited to those with: (1) documented HIV-positive status, (2) HAART receipt after July 1, 1995, with a documented HAART initiation date, (3) a CD4+ count within six months before HAART initiation and (4) at least one follow-up CD4+ count after HAART. Because they represented a distinct population, dependents of active duty personnel were not included in this analysis. Data were evaluated through February 2010.

This substudy was approved by the governing central institutional review board. The study was conducted according to the principles expressed in the Declaration of Helsinki. All study participants in the NHS provided written informed consent

### Statistical Analysis

Of 1846 patients in this analysis, 1475 (80%) had documented last negative and first positive HIV test dates, with the estimated HIV SC date calculated as the mid-point. For 371 (20%) patients, the date of the first positive but not the last negative HIV test was recorded in the study's database; the estimated SC date for these patients was imputed based upon the median time between the first positive and last negative dates for other cohort members with known and comparable first HIV positive test dates.

Baseline CD4+ count and VL were taken as the values most closely preceding the HAART initiation date within the prior 6 months. For CD4+ response curves, every six-month values were chosen based on the CD4+ count whose date most closely approximated intervals of six month follow-up from HAART initiation; CD4+ counts had to be obtained within a 3 month window of the interval date. CD4+ follow-up time was truncated at the earliest of the following: last recorded visit at which a CD4+ count was obtained; last recorded visit prior to three successive 6-month visits with missing CD4+ counts; death; or 12-year post-HAART visit.

Visual inspection of the post-HAART CD4+ response curve for all patients indicated that the CD4+ response curves were not simple linear slopes. Based on our inspection, breakpoints of 0.5 and 4.0 years post-HAART were assigned, and linear mixed effects models with splines were used to model separate CD4+ slopes for the following time periods after HAART initiation: 0 to 0.5 years; 0.5 to 4 years; and > 4 years. Random effects for intercepts and slopes were included.

Separate CD4+ response curves were generated for those initiating HAART at CD4+ "baseline" counts of ≤200, 201-349, 350-499, and ≥500 cells/mm^3^. Interactions between post-HAART time period and baseline CD4+ strata were included in linear mixed effects models to estimate and compare separate CD4+ slopes by baseline CD4+ group. Baseline characteristics between CD4+ strata were compared using chi-square tests or analysis of variance.

Unadjusted models first compared CD4+ response trajectories between the four baseline strata; multivariate models then compared baseline CD4+ strata adjusting for the following covariates: age at HAART start, gender, race/ethnicity, presence of clinical AIDS prior to HAART, baseline VL (most closely prior to HAART start), any ART prior to HAART, time from estimated HIV SC date to HAART initiation date, year of HAART start, and nadir pre-HAART CD4+ count. Clinical AIDS was defined as presence of a clinical disease (not CD4+ count) meeting the 1993 Centers for Disease Control AIDS case definition [28]. As previously defined for the NHS [29], HAART included ART regimens with drugs from two or more classes, or certain combinations of three or more nucleoside/nucleotide reverse transcriptase inhibitors (NRTI); patients on ART not meeting the HAART definition were typically on mono or dual NRTI regimens. Age was modeled as a linear spline to allow for separate linear estimates among those < 40 years and among those ≥40 years. Holm's stepdown Bonferroni method adjusted for multiple slope comparisons.

This analysis was repeated for the subset of participants defined as VL suppressors. Because VL assays with different detection limits were used during follow-up, an undetectable VL was defined as <400 copies/ml. VL suppression was defined as two consecutive undetectable VLs, with the first within 48 weeks after HAART start. Data for this subgroup were censored when two consecutive VL measurements ≥400 copies/ml were first observed.

To evaluate robustness of our main findings, additional exploratory models were constructed with additional variables added as covariates. In the first model, a time-updated variable was added to indicate whether the patient was on or off HAART at each 6-month follow-up visit. In the second model, time-updated log_10_-transformed VL (based on six-month post-HAART values) was added; the separate baseline VL covariate was removed since it is captured in the time-updated covariate. In the third model, initial HAART regimen was added, and categorized as NRTI plus protease inhibitor (PI), NRTIs plus non-nucleoside reverse transcriptase inhibitor (NNRTI), NRTIs alone, and regimens with both NNRTIs and PIs.

## Results

### Characteristics at HAART initiation

One thousand eight hundred and forty-six HIV-positive individuals met analysis inclusion criteria, with characteristics summarized in Table 1. The median length of follow-up post-HAART was 5.5 years, and median number of CD4+ count values obtained post-HAART (using six-month intervals) was 10 (interquartile range: 4, 18).

**Table 1:** Characteristics of Participants in U.S. Military HIV Natural History Study by Baseline CD4+ Strata at HAART Initiation

	**CD4+ (cells/mm**^**3**^**) at HAART start**				
	≤200	201-349	350-499	≥500	Total
	(N = 419)	(N = 580)	(N = 493)	(N = 354)	(N = 1846)
Male	397 (95%)	566 (98%)	470 (95%)	337 (95%)	1770 (96%)
Race/ethnicity					
Caucasian	169 (40%)	255 (44%)	216 (44%)	172 (49%)	812 (44%)
African American	199 (47%)	246 (42%)	211 (43%)	139 (39%)	795 (43%)
Hispanic	38 (9%)	54 (9%)	49 (10%)	27 (8%)	168 (9%)
Other	13 (3%)	25 (4%)	17 (3%)	16 (5%)	71 (4%)
Year of HAART start *					
1995-1999	311 (74%)	289 (50%)	298 (60%)	245 (69%)	1143 (62%)
2000-2003	43 (10%)	104 (18%)	97 (20%)	68 (19%)	312 (17%)
2004-2008	65 (16%)	187 (32%)	98 (20%)	41 (12%)	391 (21%)
Median age at					
HAART start (IQR) *	36 (31, 42)	34 (29, 39)	34 (28, 39)	34 (29, 39)	34 (29, 40)
CD4+ nadir (cells/mm^3^) *					
≤200	419 (100%)	83 (15%)	20 (4%)	10 (3%)	532 (29%)
201-349	0 (0%)	497 (86%)	180 (37%)	58 (16%)	735 (40%)
350-499	0 (0%)	0 (0%)	293 (59%)	96 (27%)	389 (21%)
≥500	0 (0%)	0 (0%)	0 (0%)	190 (54%)	190 (10%)
Baseline VL at HAART start (copies/ml) *					
<1000	17 (4%)	37 (6%)	59 (12%)	65 (18%)	178 (10%)
1,000-9,999	25 (6%)	111 (19%)	107 (22%)	92 (26%)	335 (18%)
10,000-99,999	156 (37%)	290 (50%)	231 (47%)	131 (37%)	808 (44%)
≥100,000	170 (41%)	117 (20%)	72 (15%)	54 (15%)	413 (22%)
Missing	51 (12%)	25 (4%)	24 (5%)	12 (3%)	112 (6%)
AIDS diagnosis prior to					
HAART *	116 (28%)	24 (4%)	19 (4%)	7 (2%)	166 (9%)
On ART prior to HAART *	277 (66%)	217 (37%)	221 (45%)	146 (41%)	861 (47%)
Median years HIV SC to					
HAART start (IQR) *	6.8 (3.5,10.1)	3.2 (1.3,7.3)	3.3 (1.4,7.2)	2.8 (1.0,7.2)	4.1 (1.4,8.4)
Median years post-HAART					
follow-up (IQR) **	4.9 (2.2,11.4)	4.5 (2.0,9.2)	6.0 (2.1,10.8)	6.1 (2.6,10.6)	5.5 (2.1, 10.5)

* P < 0.001 **P = 0.001

HAART = Highly active antiretroviral therapy; ART = Antiretroviral therapy;

VL = HIV Viral load; IQR = Interquartile range; SC = Seroconversion;

CD4+ count at HAART initiation was ≤ 200 cells/mm^3 ^for 23% of participants, 201-349 cells for 31%, 350-499 cells for 27%, and ≥500 cells for 19%. The four strata differed significantly by multiple characteristics (Table 1). Among other differences, AIDS prior to HAART, baseline VL ≥100,000 copies/ml, and longer time from SC to HAART start were all most common in those with a baseline CD4+ ≤200 cells/mm^3^.

### CD4+ response curves after HAART initiation

**Figure 1:**
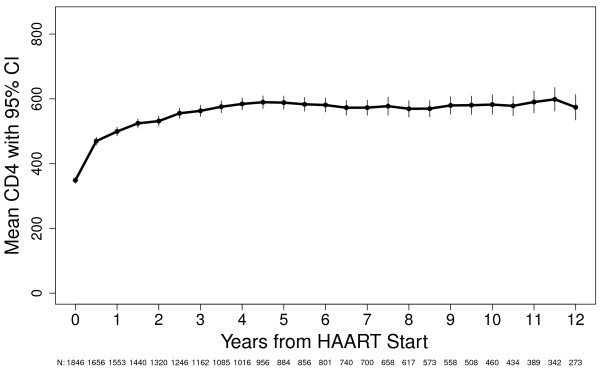
**CD4+ Response Curve After HAART Initiation for All Participants, U.S. Military HIV Natural History Study**.

Figure 1 shows the CD4+ count response after HAART initiation for all participants in this analysis. For the first 6 months after HAART initiation, the average increase in CD4+ count was 129.9 cells (95% CI 122.0, 137.8). For the second phase (0.5-4.0 years) after HAART, the average annual increase was 29.1 cells (95% CI 24.5, 33.7). For the third phase (4.0-12.0 years), the average annual change was -0.4 cells (95% CI -4.5, +3.6).

**Figure 2:**
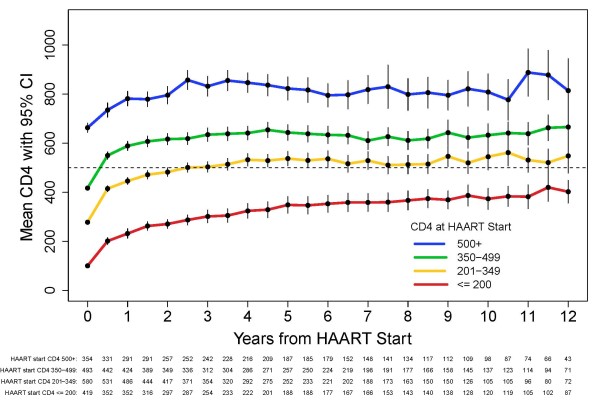
**CD4+ Response Curve After HAART by CD4+ Strata at HAART Initiation for All Participants, U.S. Military HIV Natural History Study**.

Figure 2 shows the CD4+ count response after HAART initiation by baseline CD4+ stratum. The mean CD4+ cell count at 4, 8, and 12 years post-HAART was 324, 367 and 402 (95% CI: 356, 448) for the ≤200 CD4+ cell baseline stratum; 532, 513 and 548 (95% CI: 478, 618) for the 201-349 cell stratum; 641, 611 and 666 (95% CI: 602, 729) for the 350-499 cell stratum; and 846, 799 and 814 (95% CI: 684, 945) for the ≥500 cell stratum.

The average CD4+ change and 95% CI for each of the three post-HAART time intervals are summarized in Table 2; the first time period is presented as CD4+ change per half-year; the second and third time periods present CD4+ change per year. Within all CD4+ strata, the greatest average increases (93-151 cells) were noted within the first 6 months after HAART initiation. Continued but lesser increases of 22-36 cells/year were noted during the second-phase period of 0.5-4.0 years after HAART initiation. During the third phase (>4.0 years post-HAART start), the average CD4+ count increased slightly (9 cells/year) in the lowest baseline CD4+ stratum, remained essentially unchanged in the two middle baseline strata, and decreased slightly (8 cells/year) in the highest stratum (P < 0.05).

**Table 2:** Average Change in CD4+ Count by Time Since HAART Initiation: All Participants and Viral Suppressors in U.S. Military HIV Natural History Study

CD4+ strata	**Estimated CD4+ count change and 95% CI (cells/mm**^**3**^**) by time segment (Years from HAART initiation)**		
at HAART start	0-0.5 yrs	0.5-4.0 yrs	>4.0 yrs
	(change/half-year)	(change/year)	(change/year)
≤200 cells/mm^3^			
All participants	109 (93, 126)	36 (30, 43)	8.6 (3.3, 14.0)
Viral suppressors	147 (123, 172)	66 (56, 75)	13.6 (6.0, 21.2)
201-349 cells/mm^3^			
All participants	149 (135, 162)	34 (28, 39)	3.5 (-1.3, 8.4)
Viral suppressors	171 (155, 188)	54 (48, 60)	14.4 (8.5, 20.4)
350-499 cells/mm^3^			
All participants	151 (136, 166)	22 (16, 28)	1.5 (-3.4, 6.4)
Viral suppressors	177 (159, 195)	51 (44, 57)	12.0 (6.1, 17.8)
≥500 cells/mm^3^			
All participants	93 (75, 110)	24 (17, 30)	-8.1 (-13.9, -2.4)
Viral suppressors	119 (98, 139)	56 (48, 63)	16.2 (9.8, 22.6)

* Significant (P < 0.05) differences in first-phase slopes: (1) All participants: ≤200 vs. 201-349; <200 vs. 350-499; 201-349 vs. ≥500; 350-499 vs. ≥500; (2) VL suppressors: 201-349 vs. ≥500; 350-499 vs. ≥500;

** Significant (P < 0.05) differences in second-phase slopes: (1) All participants: ≤200 vs. 350-499; (2) VL suppressors: None;

*** Significant (P < 0.05) differences in third-phase slopes: (1) All participants: ≤200 vs. ≥500; 201-349 vs. ≥500; 2) VL suppressors: None

(All P-values calculated with multiple comparisons adjustment)

HAART = Highly active antiretroviral therapy; CI = Confidence Interval

### Multivariate analysis and adjusted CD4+ slopes

In a model controlling for baseline CD4+ count and time interval after HAART start (first-, second- or third-phase), a number of other variables were significantly (P < 0.05) associated with CD4+ response (Table 3). A significantly smaller CD4+ response post-HAART occurred in those with clinical AIDS prior to HAART, a lower CD4+ nadir, a higher baseline VL, a greater number of years from HIV SC to HAART start, Hispanic ethnicity, and HAART initiation during 2000-2003 (vs. 2004-2009). Any ART prior to HAART was of borderline significance (P = 0.07).

**Table 3:** Adjusted Covariate Estimates * for CD4+ Cell Response Post-HAART for All Participants, U.S. Military HIV Natural History Study

Covariate	Estimate (95% CI)	P-value
Age at HAART start		
Effect of 10 years older if < 40 years old	4.3 (-9.3, 17.9)	0.54
Effect of 10 years older if > 40 years old	- 7.7 (-23.3, 7.9)	0.34
Female (vs. Male)	23.0 (-8.8, 54.8)	0.16
Race		
Caucasian	Reference	
African American	-7.4 (-20.5, 5.7)	0.27
Hispanic	-35.0 (-57.8, -12.2)	0.003
Other	-0.7 (-35.4, 33.9)	0.97
Clinical AIDS event prior to HAART start	-23.3 (-46.1, -0.5)	0.045
Pre -HAART nadir CD4+ count (cells/mm^3^)		
≤ 200	Reference	
201-349	57.2 (31.4, 83.0)	<.001
350-499	160.8 (129.2, 192.5)	<.001
≥ 500	236.5 (196.4, 276.6)	<.001
Baseline VL at HAART start (copies/ml) **		
< 1000	57.2 (32.5, 81.8)	<.001
1000-9999	40.2 (19.5, 60.8)	<.001
10,000-99,999	14.4 (-2.5, 31.3)	0.10
≥ 100,000	Reference	
Years from SC to HAART initiation		
≤ 1.5	Reference	
> 1.5 to 4.0	-28.3 (-47.8, -8.8)	0.005
> 4.0 to 8.5	-44.1 (-65.3, -23.0)	<.001
> 8.5	-50.6 (-73.4, -27.9)	<.001
Year of HAART initiation		
1995-1999	-12.8 (-36.6, 10.9)	0.29
2000-2003	-34.4 (-60.3, -8.4)	0.01
2004-2009	Reference	
Any ART prior to HAART start	-16.4 (-34.4, 1.6)	0.07

* Aside from baseline CD4+ count and time interval after HAART start

** Analysis adjusted for those for whom VL was missing/unknown.

HAART = Highly active antiretroviral therapy; ART = Antiretroviral therapy;

VL = HIV Viral load; SC = Seroconversion;

The numerical estimates listed in Table 3 for different levels of a specific covariate represent what the difference in post-HAART CD4+ counts would be after adjustment for all other covariates in the model. For example, after adjustment for all other covariates, a patient with a baseline VL of < 1000 copies/ml will on average have a post-HAART CD4+ count that is 57.2 cells higher than a patient with a baseline VL of ≥ 100,000 copies/ml (the referent). After adjustment, a patient infected for >8.5 years will on average have a post-HAART CD4+ count that is 50.6 cells lower than a patient infected for ≤1.5 years before HAART.

### CD4+ response curves for viral suppressors

**Figure 3:**
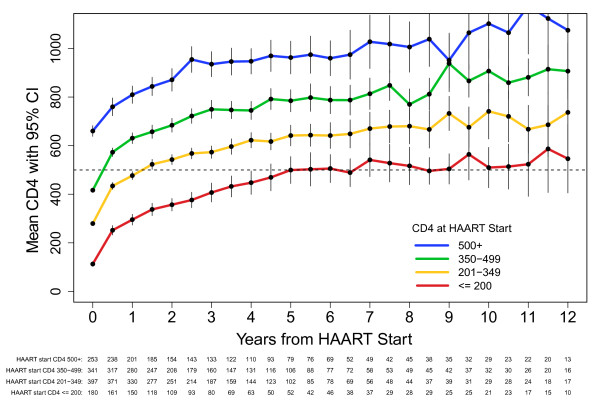
**CD4+ Response After HAART by CD4+ Strata at HAART Initiation for Viral Suppressors, U.S. Military HIV Natural History Study**.

One thousand one hundred seventy-one participants met criteria for VL suppressors. Figure 3 shows post-HAART CD4+ count responses stratified by CD4+ count at HAART initiation for VL suppressors. The mean CD4+ cell counts at 4, 8, and 12 years post-HAART for VL suppressors were 448, 517 and 546 (95% CI: 405, 687) for the ≤200 CD4+ cell baseline stratum; 622, 680 and 737 (95% CI: 561, 914) for the 201-349 CD4+ stratum; 745, 770 and 907 (95% CI: 791, 1023) for the 350-499 CD4+ stratum; and 947, 1006 and 1075 (95% CI: 820, 1330) for the ≥500 CD4+ cell stratum.

The average CD4+ change and 95% CI for each of the three post-HAART time intervals for VL suppressors are summarized in Table 2. The greatest changes were again noted within the first 6 months, followed by the 0.5-4.0 year period. For the third phase (>4.0 years) post-HAART, there were significant annual increase in all baseline strata, although only at a mean of 12-16 cells per year. Second and third phase slopes did not significantly differ for any of the baseline CD4+ strata

### Multivariate analysis for VL suppressors

In multivariate analysis for viral suppressors, factors significantly (P < 0.05) associated with a lesser CD4+ response include male sex, lower CD4+ nadir, and greater time from HIV SC to HAART start (Table 4). Clinical AIDS before HAART was of borderline significance (P = 0.057). Numerical estimates in Table 4 for different levels of a specific covariate again represent what the difference in post-HAART CD4+ counts would be after adjustment for all other covariates in the model. For example, after adjustment, a viral suppressor infected for >8.5 years will on average have a post-HAART CD4+ count that is 33.4 cells lower than a patient infected for ≤1.5 years before HAART.

**Table 4:** Adjusted Covariate Estimates* for CD4+ Cell Response Post-HAART for Viral Suppressors, U.S. Military HIV Natural History Study

Covariate	Estimate (95% CI)	P-value
Age at HAART start		
Effect of 10 years older if < 40 years old	6.3 (-9.6, 22.2)	0.44
Effect of 10 years older if > 40 years old	- 5.1 (-23.9, 13.7)	0.59
Female (vs. Male)	57.2 (14.6, 99.8)	0.009
Race		
Caucasian	Reference	
African American	9.2 (-7.3, 25.7)	0.27
Hispanic	-10.8 (-38.6, 17.1)	0.45
Other	0.4 (-40.6, 41.4)	0.98
Clinical AIDS event prior to HAART start	-32.7 (-66.4, 1.0)	0.057
Pre -HAART nadir CD4 count (cells/mm^3^)		
≤ 200	Reference	
201-349	55.3 (20.7, 89.9)	0.002
350-499	135.4 (94.6, 176.2)	<.001
≥ 500	195.1 (144.3, 245.8)	<.001
Baseline VL at HAART start (copies/ml) **		
< 1000	8.5 (-21.6, 38.6)	0.58
1000-9999	-17.8 (-43.5, 7.9)	0.18
10,000-99,999	1.3 (-19.5, 22.2)	0.90
≥ 100,000	Reference	
Years from SC to HAART initiation		
≤ 1.5	Reference	
> 1.5 to 4.0	-18.0 (-38.6, 2.6)	0.09
> 4.0 to 8.5	-27.7 (-52.0, -3.3)	0.03
≥ 8.5	-33.4 (-61.0, -5.8)	0.02
Year of HAART initiation		
1995-1999	0.0 (-22.4, 22.4)	0.99
2000-2003	5.9 (-18.1, 29.9)	0.63
2004-2009	Reference	
Any ART prior to HAART start	11.5 (-11.1, 34.0)	0.32

* Aside from baseline CD4+ count and time interval after HAART start

** Analysis adjusted for those for whom VL was missing/unknown.

HAART = Highly active antiretroviral therapy; ART = Antiretroviral therapy;

VL = HIV Viral load; SC = Seroconversion;

### Additional exploratory analyses

In the first model, the time-updated indicator of HAART use was a significant positive predictor of CD4+ response (coefficient = 95.1, 95% CI: 87.8, 102.4, P < 0.001). All significant covariates in the original adjusted model remained so, except for clinical AIDS prior to HAART, which was of borderline significance (P = 0.055). In the second model, time-updated VL after HAART start was a significant predictor of CD4+ response (coefficient = -42.6 for every log_10 _increase in VL, P < 0.001). All significant covariates in the original adjusted model remained so, except for year of HAART initiation. In the third model initial HAART regimen was added to the model. All significant covariates in the original adjusted model remained so.

## Discussion

Among HIV-positive persons starting HAART, we identified a rapid average increase of 93-151 cells during the first six months in all baseline CD4+ strata, followed by a continued average increase of 22-36 cells per year through the first four years. Among VL suppressors, these increases were even greater, with an average of 119-177 cells during the first phase, followed by an average of 51-66 cells per year during the second phase, through 4 years. For example, a patient who starts HAART with a CD4+ count of 125 cells/mm^3 ^and who maintains viral suppression will on average have an increase to about 500 cells/mm^3 ^at the end of four years.

A major purpose of this analysis was to identify whether after four years the CD4+ response continues to increase or plateaus. Among all participants, the average third-phase response was slightly positive (8.6 cells/year) in the lowest CD4+ baseline strata (≤200 cells), slightly negative (-8.1 cells/year) in the highest strata (≥500 cells), and essentially flat (with 95% CI overlapping zero) in the two middle strata. However, among VL suppressors, we identified positive average increases of approximately 12-16 cells/year, with no significant differences in third-phase slopes between any of the baseline strata. This supports the general conclusion that if viral suppression can be maintained through effective and uninterrupted HAART, a continued pattern of CD4+ count improvement may occur in most patients, irrespective of CD4+ count at HAART initiation.

There are several caveats to this overall conclusion. Although increases four years after starting HAART in viral suppressors continued to be positive, they were small. This provides support for current guidelines to start HAART at higher CD4+ levels, before severe immune suppression has occurred [1,2,30]. In addition, our analysis indicates that a variety of other factors may affect and modulate the CD4+ response curve, including nadir CD4+ cell count, AIDS prior to HAART start and pre-HAART duration of HIV infection.

Our analysis is consistent with other studies identifying nadir CD4+ count as a predictor of CD4+ cell response [31,32]. A lower nadir CD4+ count may reflect a more profound disturbance of T-cell homeostasis, with more severe immunological deficits that cannot be reversed even with HAART-induced viral suppression [33]. For example, in one analysis of response to immunization in those with normal CD4+ counts and viral suppression after more than a year, a lower CD4+ nadir before HAART predicted poorer vaccination response [34].

Our analysis also identified a clinical AIDS diagnosis preceding HAART as a predictor of a poorer CD4+ response. This may be another reflection of functional or other immune deficiencies in response to HIV infection that lead to a less robust immunologic recovery. This finding supports current recommendations to initiate HAART in all patients with a history of an AIDS-defining illness, irrespective of their current CD4+ count [1,2].

A major finding of this analysis was the strong negative effect of pre-HAART duration of HIV infection on CD4+ cell response to HAART, even after controlling for viral suppression, CD4+ count and other factors. A previous study [14] also identified duration of infection as a predictor of CD4+ response, but duration was based upon time from the first recorded HIV test rather than the entire estimated period of HIV infection, as this analysis was able to do. Potential immunopathogenic explanations for why a longer time from HIV SC to HAART start results in a more impaired capacity for immunologic recovery include decreased CD4+ cell production or excessive CD4+ cell destruction. For example, it has been proposed that CD4+ T-cell hyperactivation may persist even after HAART virologic suppression, and that this results in greater apoptotic cell death [32,33,35-37]. Our finding that both higher baseline VLs and longer duration of pre-HAART infection were predictive of poorer immunologic response suggests that long-standing high levels of viral replication may lead to persistent T-cell activation or other T-cell dysfunction which cannot be fully reversed even after HAART introduction.

This analysis has several potential limitations. First, by definition those who were followed for >5 years represent "healthy survivors"; those who died or who dropped out of the study because of illness soon after starting HAART would not be captured in the third-phase analysis. Although for the great majority of enrollees death was uncommon, it was more common among those in the lowest CD4+ cell strata. For example, a preliminary analysis of mortality among those initiating HAART at ≤200 CD4+ cells/mm^3 ^identified a 6-year death rate of 18%, compared to rates of 3%-5% for those initiating HAART at the higher CD4+ cell strata (IDCRP, unpublished data). Although many patients are still followed in the military health care system even if they are no longer on active duty after an AIDS diagnosis, some patients with advanced disease may have separated from the military system and had their health care transferred to the Veteran's Affairs or other health systems. However, even if such a healthy survivor effect did occur, we do not believe that it significantly affected our overall conclusions. Such an effect would most likely occur in the lowest (≤200 cell) CD4+ stratum, and among viral suppressors, the third-phase CD4+ increases we saw in this stratum were not significantly different from those seen in the other baseline strata. Nonetheless, the increased mortality seen in the lowest group provides additional support for current guidelines to start HAART before severe immune suppression has occurred.

Second, in this observational study, the four baseline strata were not randomized, and group differences may be due to unmeasured confounding. We tried to limit the extent of confounding by adjusting for many HIV-related factors, as well as time-dependent covariates including VL and HAART use. Analyzing our data in different ways, including through several different exploratory analyses, did not change our overall conclusions.

Third, although we adjusted for different classes of drug therapy at HAART initiation in our exploratory sensitivity analyses, we did not present data on specific ART drugs. However, this was not the intent of this analysis. Even within a given antiretroviral class, there is considerable variation depending on potency, drug-drug interactions, use of ritonavir boosting for PIs, and multiple other factors. Clinicians may select individual drugs for a HAART regimen based on a variety of factors, and information about the efficacy of specific ART drugs and regimens is best obtained through randomized trials.

Finally, this cohort is characterized by a number of specific demographic and clinical factors, and results may vary for other populations with different characteristics. For example, 96% of our study sample was male, and the median age was 36 years. Also, given the specific structured testing schedule in the military, it is likely that many patients in this cohort were diagnosed with HIV earlier than typically seen in clinical practice.

This analysis also has several strengths. First, in contrast to many other HIV cohort studies, we were able to estimate SC date and time from HIV SC to HAART start. The fact that this variable consistently emerged as a significant predictor of CD4+ response supports the importance of including this covariate in our analysis.

Second, follow-up in this analysis extended for some patients out past 8 years, considerably longer than most other observational studies. This analysis therefore provides an important contribution to the literature concerning the long-term third-phase CD4+ response to HAART, especially in those patients who maintain virologic suppression.

Third, because HIV treatment in the military is free, availability of care and access to therapy were not barriers confounding our results. In fact, viral suppression rates in this cohort have previously been reported as approaching those in clinical trials [25].

## Conclusions

Among HIV-infected persons who initiated HAART at different CD4+ levels and who were followed in some cases for over ten years, we identified a rapid followed by a more gradual increase in CD4+ cells for the first four years. After this time, among those who maintain viral suppression, our results suggest that in all strata, there will on average be a positive but small average increase of about 12-16 cells per year. However, multiple factors may influence this immunologic response, including CD4+ nadir, a preceding AIDS diagnosis, and, importantly, time from HIV infection to HAART start. Our findings strongly support the conclusion that immunologic response to HAART is maximized if treatment is started with virally suppressive therapy as early as possible.

## Competing interests

The authors declare that they have no competing interests.

## Authors' contributions

AL was lead author on planning and coordinating the analysis, and drafting interim and final versions of the manuscript. EK, PG, and LE conducted and/or provided guidance with various aspects of the statistical analysis. VM, AW, NC, AG and BA helped to implement the study, including data collection and oversight at the individual study sites at which participants were followed. EK, PG, LE, VM, AW, NC, AG, BA and MD participated in discussions concerning the design of this project, provided feedback and suggestions on interim analyses, and offered valuable input and recommendations on draft versions of this manuscript. All authors have seen and approved the final manuscript.
